# Opinion: The use of chicken IgY in the control of pandemics

**DOI:** 10.3389/fimmu.2022.954310

**Published:** 2022-08-11

**Authors:** Michael G. Wallach

**Affiliations:** ^1^University of Technology Sydney, Sydney, NSW, Australia; ^2^The Centre for Innovative Medical Research, SPARK Oceania, Sydney, NSW, Australia

**Keywords:** IgY, pandemic, COVID-19, influenza, transmission block

## Introduction

Chicken IgY when applied nasally or orally is highly effective in controlling a variety of pathogens, is extremely stable, cross protective and safe in humans (1). It is produced by laying hens as a means of protecting offspring chicks during the first few weeks of their growth allowing them time to develop their own immunity. This maternal immunity has evolved over time to protect offspring from all animal species and can be capitalized on to help protect humans in the advent of a pandemic by prophylactically lowering or blocking transmission and infection. Egg yolks contain large quantities (up to 150 mg/egg) of IgY thus making this economically affordable and feasible. Each hen produces an egg a day for up to 9 months and commercial flocks of hundreds of thousands of hens can be employed to produce enough IgY to protect millions of people. It can be formulated in various ways such as nasal drops and lozenges and strategically used to firstly protect health workers, teachers, and other frontline workers. Finally, IgY can be produced against the pathogens predicted to cause future pandemics and due to its cross protection can be stockpiled and ready to use to provide a transmission block from the early stages of a pandemic.

## Discussion

Over the course of human evolution, we have faced major pandemics caused by both viral and bacterial pathogens. The Spanish flu of 1917 was the most devastating and led to the death of 17-50 million people (and possibly much higher) within a period of 1-2 years. Of course, more recently we have experienced both influenza and coronavirus pandemics with the current one of COVID-19 causing over 6 million deaths and it is still not over.

The control of pandemics is still a major global challenge. Vaccines against pandemic-causing pathogens such as H1N1 influenza and COVID-19 viruses have only been partially effective in alleviating the level of morbidity and mortality. Indeed, as COVID-19 viral variants have emerged internationally, the effectiveness of vaccines have been reduced and new control strategies are urgently needed. Similarly with influenza, pneumonia and other infectious diseases, variations in the sequence of key proteins responsible for infection as well as pathogen resistance against vaccines and drugs have hampered our ability to control such devastating diseases.

Chicken IgY represents an attractive means of control for pandemics by acting as a prophylactic to reduce viral transmission and spread, thus lowering morbidity and mortality. IgY evolved in chickens as a means of providing maternal immunity to offspring chicks against the pathogens they are likely to encounter during their early growth period of 2-3 weeks (2). This gives the chick time to develop its own immune response and therefore it is important that maternal IgY on its own can neutralize a great variety of pathogens and their variants.

An important advantage of this approach is that due to the wide coverage of IgY against various pathogens and their variants, it is unlikely to put strong selective pressure on the virus to undergo mutation in order to escape host immunity. These mutants can often be extremely pathogenic and lead to sudden spikes in mortality and the overrunning of our hospitals as we witnessed with COVID-19. Chicken IgY on the other hand has been shown to be able to prophylactically provide protection either by using nasal drops or lozenges to coat the naso- and oro-pharynx with antibodies that block viral entry into the respiratory tract from where it can spread to the entire body. Furthermore, it was shown that these antibodies are extremely cross reactive and protective (3) when they were tested against influenza viruses H1N1, H3N2 and H5N1. Surprisingly, it was even found that IgY against H5N1, shown to provide 100% protection in an *in vivo* mouse model, was able to partially cross-protect against a Puerto Rican strain of H1N1.

Chicken IgY has many other advantages for use in pandemic control. Firstly, they can be produced in very large quantities at low cost, are stable even at room temperature (at 4°C they can be stored for several years with no loss of activity) and in that regard are particularly attractive for use in developing countries. Secondly, IgY has been shown to be safe in humans due to the fact that it does not bind complement and will not activate macrophages. This has indeed been demonstrated in a phase I clinical trial for COVID 19 (4). Finally, IgY can be stockpiled so that it is ready to use in preparing for future pandemics.

The main challenge is the implementation and use of these IgY antibodies in order to play a role in managing the transmission and spread of the pathogens that cause pandemics. In that regard, the question is how and when to use these antibodies and in which segment of the population?

Let’s start with the question of how IgY antibodies can be employed in pandemic control. There are several possible approaches including the development of nasal drops/sprays, lozenges and oral formulations, in masks, hand creams and aerosols. Thus far, we have focused on using IgY in nasal drops and lozenges for COVID-19 both of which are under development in the USA.

IgY antibodies can be produced on a large scale in commercial laying flocks or if regulatory authorities require it, in specific pathogen free (SPF) hens (5). In the case of COVID-19, hens are immunized twice at a 2 week interval using either the whole S1 antigen or the receptor binding domain (RBD) (6). Other peptides can also be used to induce in hens the production of antibodies that are very protective, cross reactive and extremely stable. These antibodies can be used in either whole, dried egg powder or in a more purified IgY preparation extracted from the egg yolks. These preparations are then formulated into a nasal drop or lozenge in the appropriate quantity. Quality control and assurance procedures are employed to ensure the safety and efficacy of the formulated IgY. These procedures include tests for antibody purity, stability and efficacy using ELISA, Western Blotting, a virus neutralization assay and sterility testing to demonstrate the absence of contaminating bacteria and viruses (7).

Results that have been obtained thus far using IgY against influenza in animal trials have shown that they are excellent at coating the mucosal surfaces and very strongly protecting against infection for up to 6-8 hours and sometimes even longer (3, 8). Of particular importance was the finding that IgY administered intranasally in mice 1 hour prior to lethal infection of influenza all survived whereas all of the control mice given normal IgY did not survive infection.

Recent results using IgY raised against the receptor binding domain of COVID-19 and administered intranasally in a hamster model demonstrated that IgY is highly protective against challenge infections based on weight gain, animal mobility and lung biopsy (9). Surprisingly this protection was found when the IgY was administered either 2 hours before infection or given as 3 doses 2, 24, and 48 hours post infection. This therapeutic effect was explained by the appearance of a small amount of IgY in the serum of the intranasally dosed hamsters.

Other recent results comparing IgY to IgY fragments produced in a phage library against COVID-19 S1 protein (IgY single chain variable region fragment, IgY-scFv) have shown that these molecules are also capable of binding to S1 in multiple sites and at a very high level of sensitivity (10). They therefore also have the potential to be used in passive immunotherapy or in a nasal/oral formulation. The advantage of such fragments is that they can be engineering for greater sensitivity and specificity than whole polyclonal IgY from eggs.

Clinical trials are underway in order to test the protective effect of IgY against COVID-19 in humans. A phase I clinical trial was carried out in Perth, Australia whereby 48 healthy adults were given purified IgY intransally for 14 days. The results of that trial have demonstrated the safety and tolerability profile of IgY raised against the receptor binding domain in a nasal drop formulation (4). It was also found that there was no significant level of IgY found in serum (cutoff of 30 ng/ml) and no increase in serum cytokines.

The next question is when to employ these nasal drop and lozenge formulations during a pandemic? It has been proposed that the focus for use of these antibodies is in protecting frontline health workers, teachers, police, etc. who are most likely to face situations of high transmission. This is particularly important when a very pathogenic virus appears in the population with a high rate of morbidity and mortality. It is clear that without extra protection against infection, health workers would not be able to carry out their jobs and get ill themselves, making it impossible to control the rapid rise in number of patients arriving at hospitals and clinics.

So the concept is simple. Every front line health worker, teacher, policeman, etc. uses the nasal drop or lozenge (lozenges would be taken after meals to avoid the intake of other proteins that may compete for the muscosal surface) just before entering an area of high transmission such as the ICU. This will act to greatly diminish the ability of the virus to infect these workers and lead to substantially lowered disease and death thus enabling their continued work with the patients. In addition, when this approach is combined with the use of vaccines, it can actually help to prevent the virus from causing any serious infection and can be looked at as an enhanced PPE. Finally, this approach will give the health workers the confidence to work in a crowded ward or ICU where a pandemic causing virus can be so deadly.

In both developed and developing countries, IgY could be produced on a very large scale in commercial flocks of hens. In the case of viral pandemics, a single protein antigen formulated in a commercially available adjuvant can be used to very rapidly produce hundreds of million or even billions of nasal drop packets and lozenges. The IgY would first be employed in hospitals, etc. and then can be used in areas of high transmission to treat the general population, particularly when expensive vaccines are not available. It is expected that on the population level, IgY would greatly help to flatten the curve, reduce mortality and increase the likelihood of developing full or partial herd immunity. As the viruses continue to circulate and IgY is being used frequently, it is expected that good immunity will develop over time reducing or eliminating the need for vaccines. Of course, when vaccines are available they should be employed to as many people in both developed and developing countries as possible.

In summary, IgY represents an attractive approach for pandemic control and preparedness. When used in a strategic manner coupled with vaccines and drugs, pandemics can be controlled and managed. Indeed, IgY offers the possibility of not having to rely on vaccines that often select for mutant strains that can lead to a situation that is worse than what one started with. IgY is cheap to produce, easy to formulate, is stable, safe and effective. In addition, due to its cross protective effect, it can be produced and stockpiled in order to be prepared for viruses or other pathogens that are likely to cause pandemics. In that manner, control can be brought about very quickly before the situation spirals out of control as we witnessed with COVID-19 at the start of the pandemic in 2020.

## Author contributions

The author confirms being the sole contributor of this work and has approved it for publication.

## Acknowledgments

I thank Dr. Isabella Hajduk for critical review and assistance.

## Conflict of interest

The authors declares that the research was conducted in the absence of any commercial or financial relationships that could be construed as a potential conflict of interest.

## Publisher’s note

All claims expressed in this article are solely those of the authors and do not necessarily represent those of their affiliated organizations, or those of the publisher, the editors and the reviewers. Any product that may be evaluated in this article, or claim that may be made by its manufacturer, is not guaranteed or endorsed by the publisher.
